# Muscle fiber-type distribution predicts weight gain and unfavorable left ventricular geometry: a 19 year follow-up study

**DOI:** 10.1186/1471-2261-6-2

**Published:** 2006-01-10

**Authors:** Jouko Karjalainen, Heikki Tikkanen, Miika Hernelahti, Urho M Kujala

**Affiliations:** 1 Unit for Sports and Exercise Medicine, University of Helsinki, Finland; 2 Central Military Hospital, Helsinki, Finland; 3 Department of Health Sciences, Faculty of Sport and Health Sciences, University of Jyväskylä, Finland

## Abstract

**Background:**

Skeletal muscle consists of type-I (slow-twitch) and type-II (fast-twitch) fibers, with proportions highly variable between individuals and mostly determined by genetic factors. Cross-sectional studies have associated low percentage of type-I fibers (type-I%) with many cardiovascular risk factors.

**Methods:**

We investigated whether baseline type-I% predicts left ventricular (LV) structure and function at 19-year follow-up, and if so, which are the strongest mediating factors. At baseline in 1984 muscle fiber-type distribution (by actomyosin ATPase staining) was studied in 63 healthy men (aged 32–58 years). The follow-up in 2003 included echocardiography, measurement of obesity related variables, physical activity and blood pressure.

**Results:**

In the 40 men not using cardiovascular drugs at follow-up, low type-I% predicted higher heart rate, blood pressure, and LV fractional shortening suggesting increased sympathetic tone. Low type-I% predicted smaller LV chamber diameters (*P *≤ 0.009) and greater relative wall thickness (*P *= 0.034) without increase in LV mass (concentric remodeling). This was explained by the association of type-I% with obesity related variables. Type-I% was an independent predictor of follow-up body fat percentage, waist/hip ratio, weight gain in adulthood, and physical activity (in all *P *≤ 0.001). After including these risk factors in the regression models, weight gain was the strongest predictor of LV geometry explaining 64% of the variation in LV end-diastolic diameter, 72% in end-systolic diameter, and 53% in relative wall thickness.

**Conclusion:**

Low type-I% predicts obesity and weight gain especially in the mid-abdomen, and consequently unfavourable LV geometry indicating increased cardiovascular risk.

## Background

Skeletal muscles consist of two main types of fiber with different metabolic and functional profiles. Slow-twitch (type I) muscle fibers have a high capacity for oxidative energy metabolism, whereas fast-twitch (type II) fibers have a high capacity for glycolytic energy production [1]. The percentage of slow-twitch type I fibers (type-I%) varies widely among individuals (range 13–96%, mean value about 50% to 60%), is mostly genetically determined [2], and is resistant to training-induced change [3,4]. Individuals with high type-I% are more suited to endurance type physical activities, whereas those with low type-I% are better endowed for speed and power type activities [1,5]. This variation in skeletal muscle fiber composition may make it easier for some people to achieve a high level of endurance fitness known to favour reduced risk of cardiovascular disease. Accordingly, former elite endurance sport athletes seem to have less cardiovascular disorders and longer life expectancy than former elite speed and strength athletes [6]. In cross-sectional studies high type-I% has been associated with high volume of physical activity, with fitness, and with a favourable lipid profile [7], while low type-I% has been found to associate with obesity [8,9], with insulin resistance [10], and with higher blood pressure [11].

As cross-sectional studies have shown that skeletal muscle fiber composition associates with several cardiovascular risk factors, we hypothesized that the proportion of slow-twitch skeletal muscle fibers could predict left ventricular (LV) structure and function. We tested this hypothesis in a follow-up study of non-athletic men in whom skeletal muscle fiber composition had been determined 19 years earlier.

## Methods

An invitation to a follow-up study was sent to 79 male participants of a 1984 baseline study designed to assess the relationships between skeletal muscle fiber composition, physical fitness and lipid profile [7,12]. The men were originally recruited from private companies in Helsinki and had volunteered for the baseline study in response to the offer of a guided exercise programme funded by their employers. Their leisure-time physical activity (LTPA) levels varied from inactive to regular jogging, but none was a competitive athlete. In 2003 four of the men had died and one had moved abroad and could not be contacted. Altogether 63 men (85% of those contacted) participated in the follow-up study including echocardiography. The study was approved by the ethics committee of the Joint Authority for the Hospital Districts of Helsinki and Uusimaa. The subjects gave informed consent. The procedures followed were in accordance with institutional guidelines.

**At baseline in 1984 **the study subjects were apparently healthy and taking no long-term medication. Skeletal muscle fiber distribution was analysed from needle samples from the lateral portion of the quadriceps femoris muscle as described in detail earlier [12]. Muscle samples were stained for actomyosin ATPase, which clearly separates the two main fiber types. A personal interview provided data on LTPA which was expressed as metabolic equivalents of oxygen consumption (MET), hours/week [7]. Height and weight were obtained to calculate the body mass index (BMI) (kg/m^2^). Weight gain during adulthood was calculated as the mean yearly change in BMI after the age of 20. Finnish men know their weight and height measured during their compulsory military service at 20 years.

**At follow-up in 2003 **the age of the 63 men ranged from 51 to 77 years. All measurements and interviews were made blinded to baseline data. The study included echocardiography and a 12-lead electrocardiogram. Blood pressure was measured twice in each individual after a five-minute rest and the value with lower mean pressure was recorded. BMI and waist/hip ratio were measured and weight gain since 20 years of age was calculated. Body fat percentage was evaluated based on four skin fold measurements (subscapular, triceps brachii, biceps brachii, and crista iliaca) with calliper [13]. Volume of LTPA, use of medication and use of alcohol were assessed by personal interview and questionnaire. Alcohol use was recorded as frequency and quantity of different beverage types, and converted into grams of absolute alcohol per day.

### Echocardiography

Studies were performed with an Acuson 128 instrument by the same experienced observer blinded to other data. LV cavity dimensions and septal and posterior wall thickness were measured from an enlarged M-mode image directly from the screen monitor. In case optimal orientation of M-mode beam could not be obtained, measurements were taken from two-dimensional images [14] with the aid of harmonic imaging software, if necessary. LV mass was calculated using the formula by Devereux [15]. LV mass and dimensions were also indexed for body surface area. Concentricity of the LV myocardium was estimated by calculating the relative wall thickness: Relative wall thickness = (septal thickness + posterior wall thickness)/LV end-diastolic diameter. LV systolic function was evaluated by calculating the percentage of fractional shortening of the LV endocardium: Fractional shortening% = (end-diastolic diameter – end-systolic diameter)/end-diastolic diameter*100.

### Statistical methods

The analyses were designed to test the hypothesis that type-I% muscle fiber composition, assessed at baseline in 1984, would predict LV structure and function measured at follow-up in 2003, and if so, to investigate the strongest mediating factors. We first compared the findings in men with low type-I% (≤60% = below median) and high type-I% (>60% = above median), and tested the differences with the t-test. Based on Pearson's bivariate correlations between the variables, multivariate linear regression models were used. Follow-up age was included first in all regression models. First, dependence of echocardiographic indices on type-I% was analysed one-by-one. Second, dependence of the different cardiac risk factors at baseline (LTPA in 1984, BMI in 1984, and adulthood weight gain until 1984) and at follow-up (obesity related variables, LTPA, systolic and diastolic blood pressure, and heart rate) on type-I% was analysed one-by-one. Third, in separate stepwise regression models baseline and follow-up cardiac risk factors were added to test which of the risk factors had the strongest impact on echocardiographic indices. All *P*-values were based on a two-sided alternative hypothesis. Statistical analyses were performed with SPSS 11.5 for Windows software.

## Results

In 1984 none of the 63 subjects were on chronic medication, but 19 years later 22 men (35%) used cardiovascular drugs (beta blockers, angiotensin-convertase enzyme inhibitors, angiotensin receptor antagonists, calcium blockers, or diuretics). Men with low type-I% used these drugs more often (47%) than those with high type-I% (23%) (*P *= 0.053, Chi-square test). Because these drugs influence LV structure and blood pressure, men using them were excluded from the final analysis. In addition to men with treated hypertension this excluded also 5 men with valve disease, sequelae of myocardial infarction, and atrial fibrillation. One man not using drugs was found to have cardiomyopathy with an ejection fraction of 33% and left bundle branch block and was excluded. Thus the final analysis included 40 men in whom type-I% varied from 23% to 88 % with a mean value of 59.6% (Table 1).

**Table 1:** Characteristics of men with low and high percentage of slow-twitch type-I skeletal muscle fibers.

	Type-I% ≤60% (n = 20)	Type-I% >60% (n = 20)	*P*
Age (years)	57.4 (4.8)	60.5 (7.2)	0.11
Type-I%	47.6 (10.6)	71.6 (7.8)	<0.001
BMI at the age of 20 (kg/m^2^)	21.7 (1.5)	21.8 (2.0)	0.90
BMI in 1984 (kg/m^2^)	24.1 (2.9)	22.4 (2.6)	0.05
BMI in 2003 (kg/m^2^)	27.5 (4.4)	24.0 (3.3)	0.007
Weight gain/year (kg/m^2^) until 2003	0.16 (0.11)	0.06 (0.06)	0.002
Waist/hip ratio in 2003	1.00 (0.005)	0.94 (0.006)	0.002
Body fat % in 2003	25.5 (5.0)	19.4 (4.2)	<0.001
LTPA in 2003 (MET)	26.5 (17.1)	42.6 (23.9)	0.019
Alcohol consumption (g/day) in 2003	14.3 (9.4)	16.7 (14.7)	0.55

Values are mean (SD). BMI = body mass index, LTPA = leisure time physical activity. Weight gain was calculated as the mean yearly change in BMI after the age of 20.

### Comparison of men with low and high type-I%

There was no difference in BMI at 20 years between the groups (Table 1), but at follow-up men with low type-I% had gained more weight being 13.1 kg heavier on average. LTPA was 61% higher at follow-up in the high type-I% group and did not differ significantly from LTPA at baseline in either group. Table 2 shows the echocardiograhic findings in men with low and high type-I%. LV mass, whether indexed for body surface area or not, did not differ between the groups. Men with low type-I% had smaller chamber diameters especially if indexed for body surface area, but their mean wall thickness did not differ. This resulted in 11% greater relative wall thickness and more concentric LV myocardium. In addition to higher blood pressure, men with low type-I% also had signs of more hyperdynamic systolic function of the LV; fractional shortening was 15% and heart rate 16% higher.

**Table 2:** Cardiovascular findings at follow-up in men with low and high percentage of slow-twitch type I skeletal muscle fibers.

	Type I fibers ≤60% (n = 20)	Type I fibers >60% (n = 20)	*P*
Heart rate (beats/min)	64 (10)	55 (9)	0.006
Systolic blood pressure (mmHg)	144 (21)	138 (19)	0.35
Diastolic blood pressure (mmHg)	91 (8)	85 (7)	0.016
LV end-diastolic diameter (mm)	51.6 (4.6)	53.9 (4.3)	0.13
LV end-diastolic diameter (mm/m^2^)	25.1 (3.1)	28.3 (3.1)	<0.001
LV end-systolic diameter (mm)	31.7 (4.1)	35.8 (3.9)	0.003
LV end-systolic diameter (mm/m^2^)	15.4(2.7)	18.8 (2.5)	<0.001
Mean LV wall thickness (mm)	10.8 (1.7)	10,2 (1.2)	0.25
Mean LV wall thickness (mm/m^2^)	5.2 (0.7)	5.4 (0.6)	0.42
LV mass (g)	215 (52)	216 (52)	0.95
LV mass (g/m^2^)	104 (22)	112 (25)	0.22
Relative wall thickness	0.42 (0.009)	0.38 (0.05)	0.07
LV fractional shortening (%)	38.7 (4.3)	33.7 (3.7)	<0.001

Values are mean (SD). LV = left ventricle

### Type-I% as a predictor of echocardiographic indices

**Figure 1:**
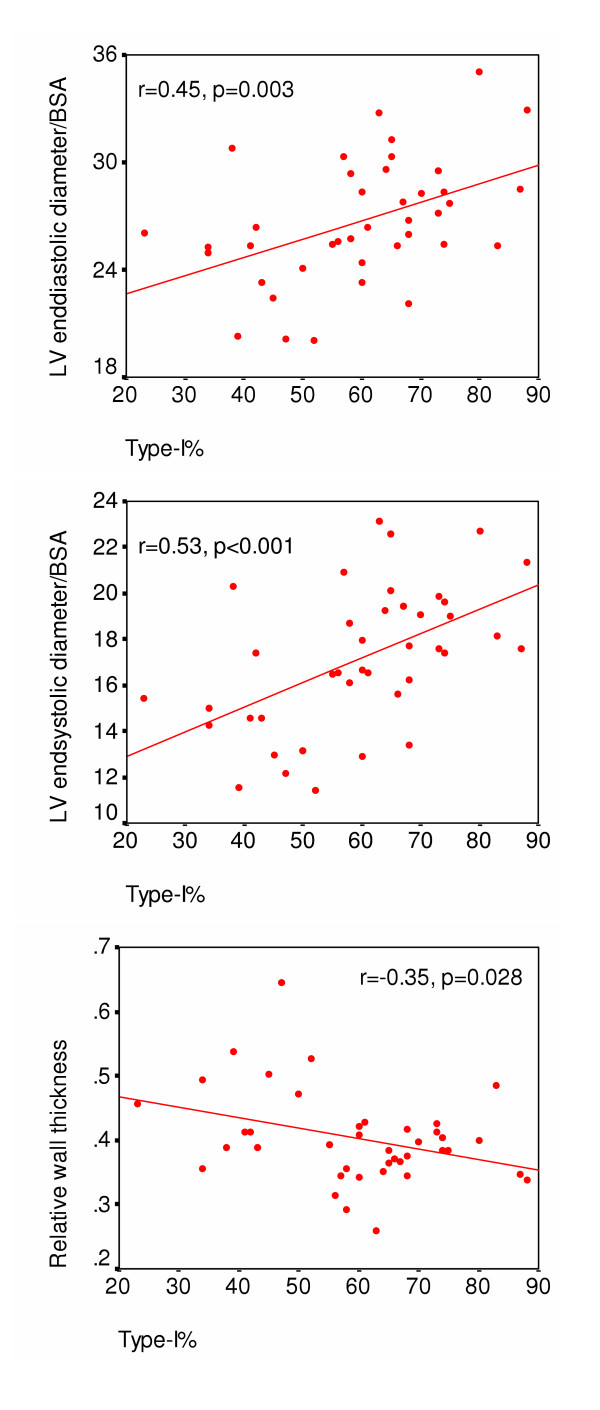
Scatterplots showing the association of percentage of type-I fibers at baseline with left ventricular dimensions indexed for body surface area and with relative wall thickness at follow-up.

In regression models low type-I% predicted smaller LV with increased relative wall thickness (concentric remodeling) and greater fractional shortening. (Table 3, Fig. 1).

**Table 3:** Percentage of slow-twitch type-I muscle fibers as a predictor of echocardiographic indices at follow-up.

Dependent variable	Regression coefficient B (95% confidence interval)	*P*-value	R square
LV end-diastolic diameter (mm/m^2^)	0.093 (0.024 to 0.162)	0.009	0.23
LV end-systolic diameter (mm/m^2^)	0.097 (0.039 to 0.156)	0.002	0.30
LV mean wall thickness (mm/m^2^)	-0.002 (-0.017 to 0.012)	0.768	
Relative wall thickness	-0.002 (-0.003 to 0.000)	0.034	0.12
LV mass (g/m^2^)	-0.055 (-0.581 to 0.470)	0.833	
Fractional shortening (%)	-0.150 (-0.240 to -0.059)	0.002	0.30

Results are adjusted for age.

### Type-I%, age, LTPA, and weight gain at baseline

Age did not associate with type-I%. Lower type-I% predicted greater weight gain after the age of 20 until baseline in 1984, explaining 27% of its variation. Type-I% also associated with baseline LTPA, explaining alone 39% of its variation and together with age 52% (Table 4). Type-I% was the only significant predictor of weight gain if both type-I% and LTPA were included as independent variables in the same model.

**Table 4:** Percentage of slow-switch type-I muscle fibers as a predictor of cardiac risk factors at baseline and at follow-up

Dependent variable	Regression coefficient B (95% confidence interval)	*P*-value	R square
Baseline 1984			
Body mass index (kg/m^2^)	-0.085 (-0.144 to -0.027)	0.005	0.19
Weight gain (kg/m^2^/year)	-0.005 (-0.007 to -0.002)	0.001	0.27
Physical activity (MET)	1.098 (0.588 to 1.607)	<0.001	0.52
Follow-up 2003			
Body mass index (kg/m^2^)	-0.134 (-0.218 to -0.051)	0.002	0.23
Weight gain (kg/m^2^/year)	-0.003 (-0.005 to -0.001)	0.001	0.29
Waist/hip ratio	-0.002 (-0.003 to -0.001)	0.001	0.30
Body fat (%)	-0.223 (-0.316 to -0.130)	<0.001	0.45
Physical activity (MET)	0.823 (0.409 to 1.238)	<0.001	0.31
Systolic blood pressure (mmHg)	-0.460 (-0.858 to -0.061)	0.025	0.23
Diastolic blood pressure (mmHg)	-0.261 (-0.419 to -0.103)	0.002	0.24
Heart rate (beats/min)	-0.322 (-0.542 to -0.102)	0.005	0.20

Results are adjusted for age. Age was a significant predictor of physical activity in 1984 (B = 1.937, 95%CI 0.693 to 3.182, *P *= 0.003), and systolic blood pressure at follow-up (B = 1.397, 95%CI 0.424 to 2.370, *P *= 0.006)

Weight gain was calculated as mean yearly change in body mass index after the age of 20.

### Type-I% and cardiac risk factors at follow-up

**Figure 2:**
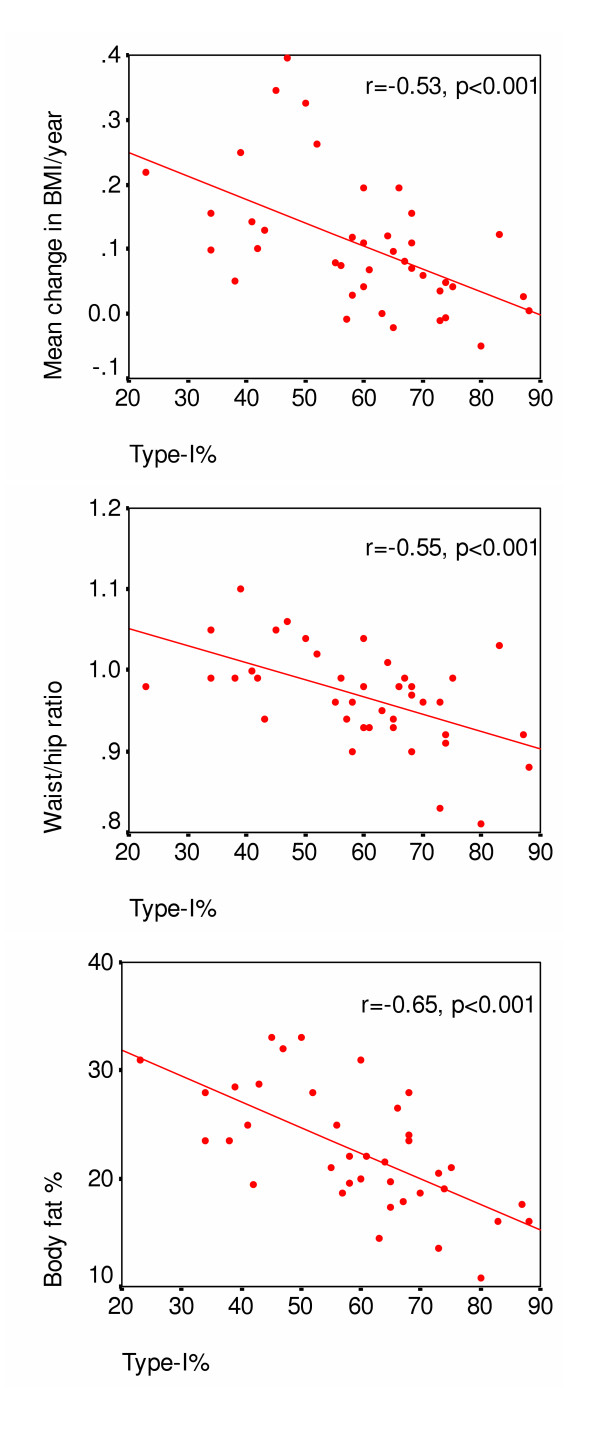
Scatterplots showing the association of percentage of type-I fibers with weight gain in adulthood, with body fat percentage, and with middle body obesity at follow-up.

Pearson's bivariate correlations showed that type-I% had close interrelations with LTPA in 2003 (R = 0.56, *P *< 0.001), and with variables related to obesity (for BMI R = -0.47, *P *= 0.002; for waist/hip ratio R = -0.55, *P *=< 0.001; for body fat percentage R = -0.65, *P *< 0.001; and for weight gain R = -0.52, *P *= 0.001). All obesity-related variables were predicted by type-I% in regression analysis adjusted for age (Table 4, Fig. 2). Body fat percentage associated also with LTPA in 2003. Low type-I% also independently predicted higher diastolic blood pressure and, in addition to age, higher systolic blood pressure.

### Type-I%, cardiac risk factors and echocardiographic indices

**Figure 3:**
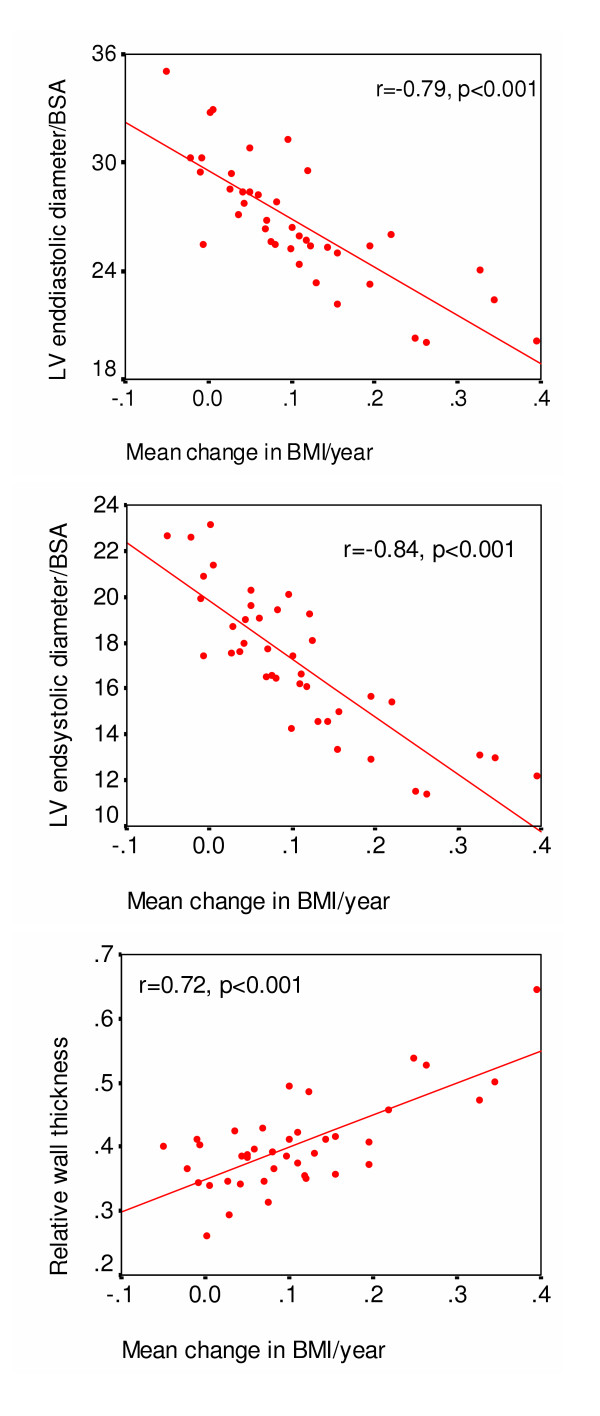
Scatterplots showing the association of weight gain in adulthood with left ventricular dimensions indexed for body surface area and with relative wall thickness.

When baseline cardiac risk factors (LTPA 1984, and weight gain 1984 or BMI 1984) were added stepwise into the model, adulthood weight gain 1984 significantly improved the explanatory rate of the model for LV diastolic (*P *= 0.006, R^2 ^= 0.38) and systolic (*P *= 0.004, R^2 ^= 0.45) dimensions and relative wall thickness (*P *= 0.001, R^2 ^= 0.37). Type-I% remained, however, an independent predictor of systolic LV function (*P *= 0.002, R^2 ^= 0.30). The cross-sectional impact of follow-up risk factors on echocardiographic indices is shown in Table 5. Weight gain until 2003 had a strong negative association with indexed LV dimensions and a positive association with relative wall thickness and thus with concentric remodeling (Fig. 3). The strongest predictor of LV fractional shortening was body fat percentage.

**Table 5:** Predictors of echocardiographic indices, with follow-up risk factors included stepwise into the model. Percentage of type-I fibers, blood pressure, physical activity, heart rate, and one obesity-related variable were the independent variables.

Dependent variable	Strongest follow-up variables entering the model	Regression coefficient B (95% confidence interval)	*P*-value	R square
LV end-diastolic diameter (mm/m^2^)	Weight gain 2003	-25.64 (-33.74 to -17.53)	<0.001	0.64
LV end-systolic diameter (mm/m^2^)	Weight gain 2003	-22.99 (-29.40 to -16.59)	<0.001	0.72
LV mean wall thickness (mm/m^2^)	None			
Relative wall thickness	Weight gain 2003	0.526 (0.333 to 0.718)	<0.001	0.53
LV mass (g/m^2^)	None			
Fractional shortening (%)	Body fat %	0.603 (0.347 to 0.859)	<0.001	0.57

Results are adjusted for age. LV = left ventricle.

Weight gain was calculated as mean yearly change in body mass index after the age of 20

We performed similar regression analyses also in the whole study group including the men using cardiovascular drugs with comparable results: Type-I% predicted LV chamber diameters and systolic function (*P *< 0.001–0.009), but not LV wall thickness or LV mass. Type-I% also predicted follow-up LTPA (*P *< 0.001) and obesity related variables (*P *= 0.002–0.014). After including the follow-up risk factors in the regression models weight gain was again the strongest predictor of LV diameters and relative wall thickness (in all *P *< 0.001) but also type-I% remained a significant predictor for LV endsystolic diameter (*P *= 0.004) and fractional shortening (*P *< 0.001).

## Discussion

Skeletal muscles, representing 35–45% of body mass, play a central role in whole-body energy metabolism [1]. Our follow-up study shows that the fiber composition of skeletal muscles, which dictates their metabolic and oxidative profile, is profoundly associated with cardiovascular risk factors and consequently with unfavorable LV geometry. All such disadvantageous findings seem to accumulate in men with a low percentage of slow-twitch type I muscle fibers.

In our study type-I% at baseline predicted follow-up BMI, waist/hip ratio, fatness and weight gain in adulthood, confirming earlier cross-sectional findings [8,9]. Interestingly, our subjects' BMI at the age of 20 had no relationship with the type-I%. However, 12–38 years later at baseline in1984 this association was already clear and at follow-up in 2003 even stronger (Table 1 and 4). Greater weight gain in subjects with low type-I% may be linked to the hampered ability of skeletal muscle to oxidize lipid [16]. Weight loss interventions have been more successful in obese women with higher type-I% compared with those having low type-I% [9]. Further, lean men with high type-I% consume more fat during similar exercise intensity than obese men with lower type-I% [8]. In our study type-I% also associated closely with volume of LTPA both at baseline and follow-up, which would partly explain the lesser extent of fatness in high type-I% men. Thus it would seem that obesity resistance in high type-I% subjects is due to both the metabolic properties of the muscle and to the propensity for high level physical activity.

Low type-I% also independently predicted higher blood pressure as we have reported in detail earlier [17]. Further, heart rate and fractional shortening of the left ventricle were also higher in low type-I% men, largely explained by greater obesity-related variables in these men. Together, these findings may reflect increased sympathetic tone in low type-I% subjects, as has also been suggested by others [18].

Low type-I% predicted concentric remodeling of the left ventricle. The impact of low type-I% was, however, mostly mediated by an increase in BMI (Table 4 and 5). Cross-sectional studies have earlier associated obesity with concentric LV remodeling in young women [19] and in healthy adolescents [20], while eccentric LV hypertrophy has been described in morbid obesity [21]. We found a strong association between LV geometry and weight gain (Fig. 3) although in most of our overweight men their obesity was quite modest. This relationship was clear-cut despite the exclusion of men with drug therapy for cardiovascular disorders. Concentric LV remodeling is a sign of arterial stiffening [22] and associates with carotid artery intima-media thickness [23], a highly heritable surrogate marker of atherosclerosis. It is believed that variation in each of multiple cardiovascular candidate genes exerts a small effect on the development of atherosclerosis [24].

Genes regulating the expression of skeletal muscle fibers [4] seem to be such candidates. Indeed, Wang and coworkers [25] genetically induced a fiber switch to type I in mice by targeted expression of an activated form of peroxisome proliferator-activated receptor δ in skeletal muscle, a major transcriptional regulator of fat burning. Compared to littermates these mice were resistant to obesity and had high endurance fitness, like our men with high type-I%. Further, Wisloff and coworkers [26] found that cardiovascular risk factors emerged in rats after artificial selection for low intrinsic aerobic capacity. Low capacity rats had lower peroxisome profilerator-activated receptor levels than high capacity rats in their soleus muscle, known to be rich in type I fibers; they also weighed more, had increased visceral weight, higher blood pressure and a tendency to shorter and wider myocardial cells. These observations are consistent with our findings in men with low type-I%.

The limited sample size study caused a statistical power limitation in designing multivariate analyses. Only the strongest pathways mediating associations between muscle fiber type and echocardiographic measurements could be identified: of these, body fat accumulation/weight gain was predominant. Our study subjects were white men, thus our findings may not be applicable to other ethnic groups or women. Another limitation of our study was that the measurement of muscle fiber distribution was not repeated at follow-up. Thus we have no direct evidence that the distribution of fiber types was the same as at baseline. However, aging has not been found to change the percentage of type I fibers [27]. We studied only the main muscle fiber types I and II.

Type II fibers can be further classified by ATPase staining into IIa, IIb, and IIx fibers. Resistance- and endurance training, aging and obesity may induce transformation between subtypes of II fibers, and in small mammals training may also induce a switch to type I muscle fibers [4,28]. Humans do not, however, exhibit increased expression of type-I muscle fibers with either endurance or strength training [3,4,29]. Nevertheless, they do exhibit significant increases in oxidative capacity in all fiber types with endurance training [4]. Therefore we believe that endurance training is especially important for subjects born with low type-I%. This could assist in weight control and subsequently decrease cardiovascular risks.

## Conclusion

We conclude that low proportion of type I muscle fibers predicts obesity and weight gain especially in the mid-abdomen, and consequently unfavorable LV geometry, concentric remodeling. Skeletal muscle fiber composition seems to be an important background contributor to cardiovascular risk factors.

## Competing interests

The author(s) declare that they have no competing interests.

## Authors' contributions

J. Karjalainen made echocardiographic studies, analyzed the data, and drafted the paper, H. Tikkanen designed the study and collected the baseline data, M. Hernelahti collected the follow-up data and contributed to editing of the paper, U. Kujala contributed to analyzing the data and writing the paper.

## Pre-publication history

The pre-publication history for this paper can be accessed here:


